# Topology of diffusion changes in corpus callosum in Alzheimer's disease: An exploratory case-control study

**DOI:** 10.3389/fneur.2022.1005406

**Published:** 2022-11-30

**Authors:** Sumeet Kumar, Alberto De Luca, Alexander Leemans, Seyed Ehsan Saffari, Septian Hartono, Fatin Zahra Zailan, Kok Pin Ng, Nagaendran Kandiah

**Affiliations:** ^1^National Neuroscience Institute, Singapore, Singapore; ^2^Duke-NUS Graduate Medical School, Singapore, Singapore; ^3^Image Sciences Institute, UMC Utrecht, Utrecht, Netherlands; ^4^Lee Kong Chian School of Medicine, Nanyang Technological University, Singapore, Singapore

**Keywords:** Alzheimer's disease, diffusion tensor imaging, white matter tract integrity, corpus callosum, magnetic resonance imaging (MRI), diffusion kurtosis imaging, axial kurtosis

## Abstract

**Aim:**

This study aims to assess the integrity of white matter in various segments of the corpus callosum in Alzheimer's disease (AD) by using metrics derived from diffusion tensor imaging (DTI), diffusion kurtosis imaging (DKI) and white matter tract integrity model (WMTI) and compare these findings to healthy controls (HC).

**Methods:**

The study was approved by the institutional ethics board. 12 AD patients and 12 HC formed the study population. All AD patients were recruited from a tertiary neurology memory clinic. A standardized battery of neuropsychological assessments was administered to the study participants by a trained rater. MRI scans were performed with a Philips Ingenia 3.0T scanner equipped with a 32-channel head coil. The protocol included a T1-weighted sequence, FLAIR and a dMRI acquisition. The dMRI scan included a total of 71 volumes, 8 at b = 0 s/mm^2^, 15 at b = 1,000 s/mm^2^ and 48 at b = 2,000 s/mm^2^. Diffusion data fit was performed using DKI REKINDLE and WMTI models.

**Results and discussion:**

We detected changes suggesting demyelination and axonal degeneration throughout the corpus callosum of patients with AD, most prominent in the mid-anterior and mid-posterior segments of CC. Axial kurtosis was the most significantly altered metric, being reduced in AD patients in almost all segments of corpus callosum. Reduced axial kurtosis in the CC segments correlated with poor cognition scores in AD patients in the visuospatial, language and attention domains.

## Introduction

The corpus callosum is the largest commissural white matter tract connecting homotopic and heterotopic cortical areas across the two cerebral hemispheres. Commissural fibers allow transfer of information and coordination between the cerebral hemispheres. They are important in performance of higher cognitive tasks such as learning, recall of memory, visuo-spatial and executive functions (1–3). Many of these same functions are adversely affected in Alzheimer's disease (AD).

Although the hallmark histopathological findings of AD are extracellular amyloid plaques and intraneuronal neurofibrillary tangles seen in the cortex or gray matter, increasing attention is being drawn to the mechanistic role of white matter changes in the development of Alzheimer's disease based on autopsy and pathological findings of myelin loss, decrease in oligodendrocytes and activation of microglia (4–6). Recently, imaging and histopathological studies have suggested the involvement of white matter at macro- and microstructural levels in the cascade of AD pathology and in the development of clinical cognitive impairment in AD patients (7, 8). Some neuropathological studies have implicated the loss of interhemispheric connections in the corpus callosum in the pathogenesis of AD (9–12).

The organization and microstructural properties of the brain white matter can be studied with diffusion MRI, a technique sensitive to the motion of water molecules at the microscopic scale. By acquiring brain data with a moderate diffusion weighting (or b-value, b), e.g., 700 < b < 1,200 s/mm repeated for 6+ directions distributed on a sphere, the diffusion process can be characterized as a Gaussian process with diffusion tensor imaging (DTI). The main spatial direction of diffusion as well as well-established surrogate markers of brain microstructure such as the mean diffusivity (MD) and fractional anisotropy (FA) can be determined. Although DTI metrics, particularly FA, are sensitive to a change in microstructure in white matter, DTI has limited specificity to differentiate and characterize the pathological processes occurring in the white matter microstructure in AD (13).

The diffusion kurtosis imaging (DKI) model is an extension of DTI, which takes into account non-Gaussian water diffusion occurring in the brain which may be due to presence of cell membranes and microscopic barriers to water diffusion, including myelin sheaths around the axons, cell membranes and neural filaments (14). DKI requires the acquisition of additional data as compared to DTI (e.g., at least 2 diffusion weightings and 21+ unique measurements), but provides more sensitive information about microstructural changes in the tissues. As with DTI metrics, however, DKI estimates are a surrogate marker of brain microstructure and not specific biophysical measures. To mitigate this limitation, Fieremans et al. introduced the white matter tract integrity model (WMTI), a technique to extract metrics related to the intra-cellular space, extra-cellular space and axonal water fraction under the assumption that white matter consists of two non-exchanging compartments that can be modeled as tensors (15). Microstructural changes in the different compartments of white matter, intra and extra axonal compartment, can be thus derived from DKI metrics to provide a biophysical basis for the pathological changes, as shown by Fieremans et al. (16).

This study aims to assess the integrity of white matter in various segments of the corpus callosum in AD by using metrics derived from DTI, DKI, and WMTI and compare these findings to healthy controls (HC). By analyzing the diffusion changes, the aim is to understand the biophysical changes in the corpus callosum and whether these changes reflect Wallerian degeneration or demyelination in each segment. The correlation of the cognitive function of AD patients with microstructural changes in the white matter tract integrity in the corpus callosum will be explored.

## Methods

### Subject recruitment

Twenty-four study participants, 12 AD patients (3 males, 9 females; mean age 69.06 years) and 12 HC (3 males, 9 females; mean age 63.76 years), were prospectively recruited. All AD patients were recruited from a tertiary neurology memory clinic. The diagnosis of AD dementia was made by neurologists using the Diagnostic and Statistical Manual of Mental Disorders, 5th Edition (DSM-5) (17) and the National Institute on Aging and Alzheimer's Association (NIA-AA) criteria (18). Exclusion criteria included presence of any other neurological disease (such as past stroke, epilepsy, head injury) or psychiatric illness. The HC, age and gender matched, were recruited from the community. All recruited individuals underwent a comprehensive neuropsychological assessment and an MRI brain scan. The study was approved by the Institutional Review Board. Written consent was obtained from all study participants.

### Neuropsychological assessments

A standardized battery of neuropsychological assessments was administered to the study participants by a trained rater. Cognitive scores were evaluated using Mini-Mental State Examination (MMSE), Montreal Cognitive Assessment (MoCA) and repeatable battery for the assessment of neuropsychological status (RBANS). Domains tested in the RBANS includes (1) immediate memory consisting of List Learning and Story Memory, (2) visuospatial construction consisting of Figure Copy and Line Orientation, (3) language consisting of Picture Naming and Semantic Fluency, (4) attention using Digit Span and Coding and (5) delayed memory consisting of List Recall, List Recognition, Story Recall and Figure Recall.

### MRI acquisition

Acquisitions were performed with a Philips Ingenia 3.0T scanner equipped with a 32-channel head coil. The protocol included a T1-weighted sequence, fluid attenuated inversion recovery (FLAIR) and a dMRI acquisition. The imaging parameters of the three sequences are reported in Table 1. The dMRI scan included a total of 71 volumes, 8 at b = 0 s/mm^2^, 15 at b = 1,000 s/mm^2^ and 48 at b = 2,000 s/mm^2^.

**Table 1:** MR imaging parameters.

**Sequence**	**dMRI**	**3D Sagittal FLAIR**	**3D Axial T1W**
FOV (mm)	224 × 224	240 × 240	256 × 256
Voxel size (mm)	2 × 2	1.2 × 1.2	1 × 1
Matrix	112 × 112	200 × 200	256 × 256
Slice thickness (mm)	2	0.6	1
TR	8,266	4,800	7.2
TE	107	344	3.3
Flip angle	90	40 (refocussing angle)	8

dMRI data was pre-processed with a combination of in-house scripts and ExploreDTI v.4.8.6 (19) using MATLAB R2018a. The pre-processing included signal drift correction, MP-PCA denoising, and combined motion, eddy currents correction and susceptibility correction including b-matrix rotation (20–22). The susceptibility correction was performed by computing the non-linear registration of the FA image to the T1-weighted image and constraining a b-spline based deformation only on the antero-posterior direction.

The DKI fit was performed using the REKINDLE approach (23) and constraining the mean kurtosis (MK) within physiologically plausible values, e.g., 0 < MK < 3. The axial diffusivity (Dax), radial diffusivity (Drad), mean diffusivity (MD), axial kurtosis (AK) and kurtosis anisotropy (KA) were derived from the DKI fit. Subsequently, we applied the *post-hoc* WMTI model previously introduced from Fieremans et al. which attempts a biophysical interpretation of the DKI metrics. The model is based on a two-compartment formulation without exchange, and quantifies axonal water fraction [AWF], axial [AxEAD] and radial [RadEAD] diffusivity in the extra-axonal space. Radial intra axonal diffusivity [RadIAD] and axial intra axonal diffusivity [AxIAD] are also determined.

**Figure 1:**
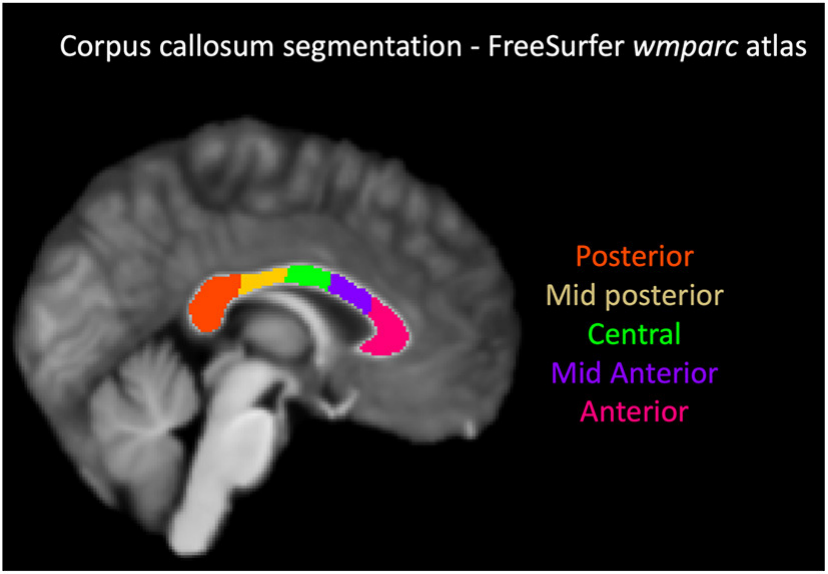
Corpus callosum segmentation with FreeSurfer wmparc atlas. From the anterior to posterior aspect, the segments were labeled and color coded as follows: anterior-magenta, mid anterior-purple, central-green, mid posterior-yellow, posterior-orange.

**Figure 2:**
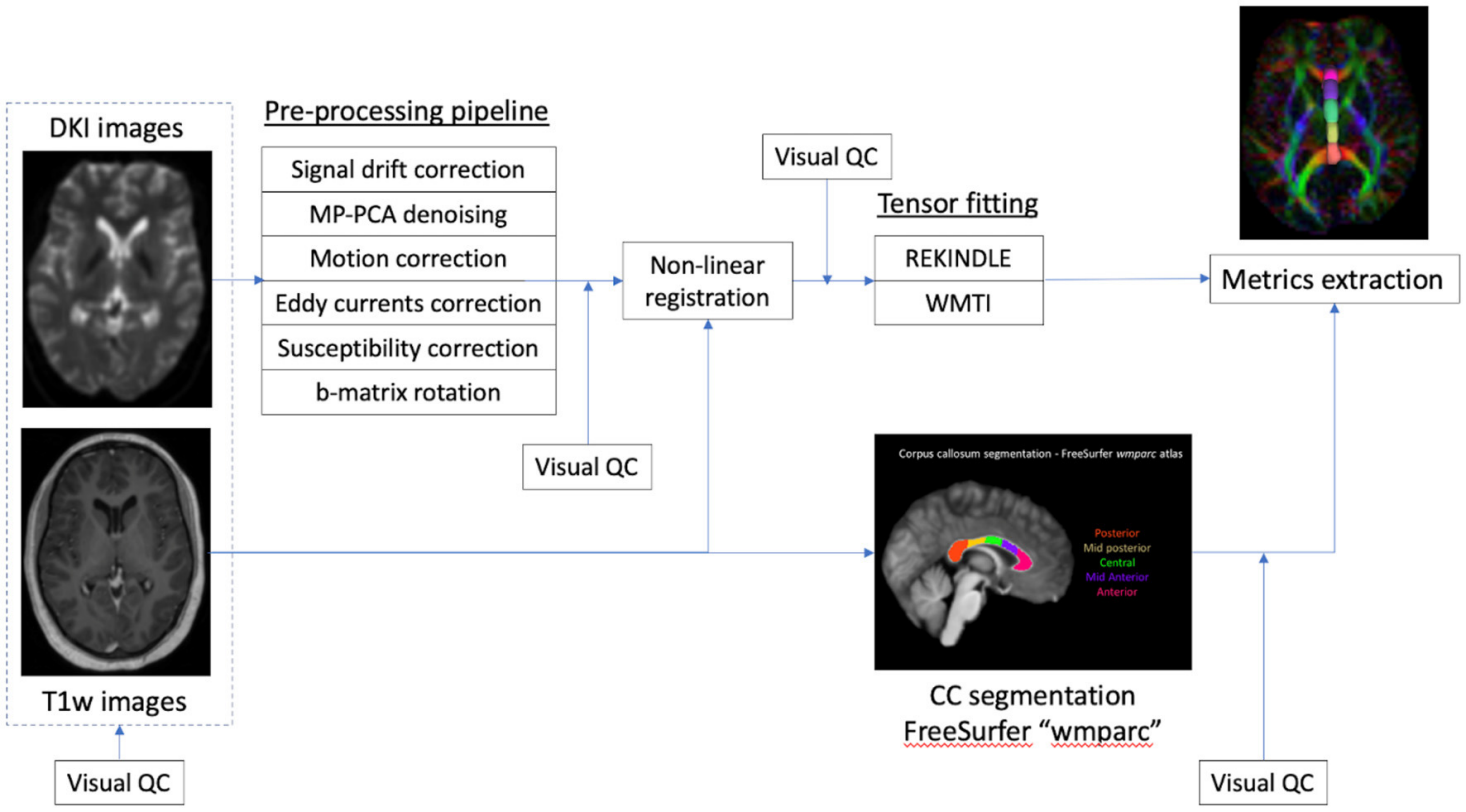
DTI post-processing pipeline.

The T1-weighted image was pre-processed with FreeSurfer v5 (24), and the corpus callosum segmented in 5 segments using the “wmparc” atlas, as shown in Figure 1. The atlas-based segmentation divides the corpus callosum into 5 equal segments from anterior to posterior and labels them as anterior, mid-anterior, central, mid-posterior and posterior. The overall post-processing pipeline as shown in Figure 2.

### Statistical analysis

The demographic characteristics of the study participants (age and gender) and their cognitive scores (MMSE, MoCa and domains in RBANS: memory, visuospatial function, attention and language) were reported as descriptive statistics (mean and standard deviation for continuous variables; frequency and percent for categorical variables). The continuous variables were compared between AD and HC using Mann Whitney U test. DTI and white matter tract integrity [WMTI] parameters were presented as mean and standard deviation by five regions (CC anterior, CC central, CC mid-anterior, CC mid-posterior, CC posterior) and compared between AD and HC. The mean differences and 95% confidence intervals of the differences were tested for significance using two independent sample *t*-test. The normality assumption of the residuals was assessed *via* quantile-quantile (QQ) plot and no major deviation from normality assumption was observed. As this is an exploratory study, false discovery rate (FDR) approach was used to adjust for multiple comparison where the number of tests was set as number of regions x number of diffusion quantities, and the adjusted *p*-values were calculated. The log(*adj p value*) were plotted for the above comparison to visually identify the potential differences between ADs and HCs. Correlation of the statistically significant DKI parameters and cognitive domains was performed using Spearman's correlation coefficient by AD and HC groups. FDR threshold was controlled at 5% and 10%. Statistical analysis was performed using SAS software version 9.4 for Windows (Cary, NC: SAS Institute Inc.).

## Results

### Patient demographics

Twelve AD patients and age and gender matched 12 HCs were included in this study. Each group included 3 men and 9 women with mean age of 69.06 ± 8.56 and 63.76 ± 6.05 in the AD and HC group, respectively. The mean global cognition scores as measured by MMSE and MOCA scores of AD group were 18.91 ± 4.08 and 15.58 ± 4.76, respectively and that of the HC group were 28.17 ± 1.90 and 26.75 ± 3.33, respectively. The results of the neuropsychological assessment in the cognitive domains (memory, visuo spatial function, attention and language), demographical characteristics and clinical assessment of the subjects of both groups are summarized in Table 2.

**Table 2:** Clinical demographics of the subjects in the study.

**Variable**	**AD**	**HC**	*p*-value*
	**(*n* = 12)**	**(*n* = 12)**	
Male/Female	3/9	3/9	–
**Age (years)**			–
Mean ± standard deviation	69.06 ± 8.56	63.76 ± 6.05	
Median (Minimum–Maximum)	71.44 (51.94–83.98)	65.21 (52.6–75.5)	
**MMSE**			
Mean ± standard deviation	18.91 ± 4.08	28.17 ± 1.90	< 0.001
Median (Minimum–Maximum)	18 (13–27)	28 (23–30)	
**MOCA**			< 0.001
Mean ± standard deviation	15.58 ± 4.76	26.75 ± 3.33	
Median (Minimum–Maximum)	17.5 (9–24)	28 (19–30)	
**Delayed Memory**			< 0.001
Mean ± standard deviation	52.25 ± 13.21	105.08 ± 14.43	
Median (Minimum–Maximum)	48 (40–84)	105.5 (81–126)	
**Visuo spatial function**			0.004
Mean ± standard deviation	81.17 ± 19.83	106.08 ± 19.64	
Median (Minimum–Maximum)	85.5 (50–116)	108.5 (64–126)	
**Attention**			0.001
Mean ± standard deviation	76.25 ± 18.97	105.42 ± 13.69	
Median (Minimum–Maximum)	77 (49–109)	104.5 (82–135)	
**Language**			< 0.001
Mean ± standard deviation	71.75 ± 15.67	95.17 ± 10.22	
Median (Minimum–Maximum)	76.5 (44–88)	92 (83–113)	
**Fazekas score (periventricular)**			0.435
Mean ± standard deviation	1.75 ± 0.97	1.33 ± 0.49	
Median (Minimum–Maximum)	1 (1–3)	1 (1–2)	
**Fazekas score (deep white matter)**			0.238
Mean ± standard deviation	1.42 ± 0.67	1 ± 0.60	
Median (Minimum–Maximum)	1 (1–3)	1 (0–2)	

^*^Two groups are compared using Mann-Whitney *U*-test.

AD, Alzheimer's disease; HC, healthy controls; MMSE, Mini-Mental State Examination; MoCA, Montreal Cognitive Assessment.

### MRI

FLAIR and T1W MR images of the brain were visually inspected by a neuroradiologist to confirm the absence of a structural brain lesion and white matter hyperintensities (WMH). WMH were visually graded by the Fazekas scale (25) and detailed in Supplementary Table 1.

#### DTI/DKI findings in the segments of corpus callosum

**Figure 3:**
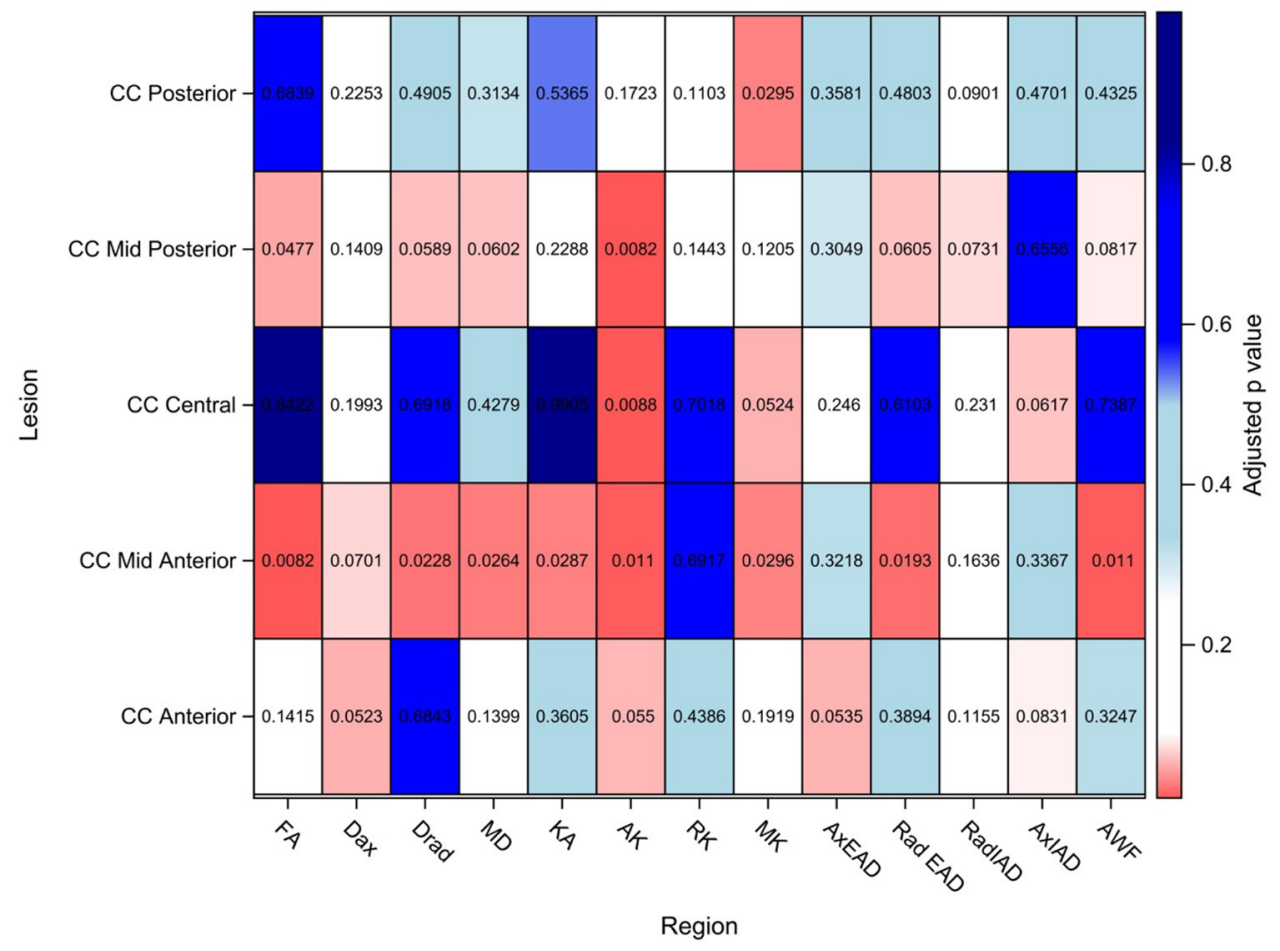
DTI/DKI/WMTI findings in the segments of corpus callosum. Values shown here are adjusted *p*-values after adjusting for multiple comparison. Adjusted *p*-value < 0.10 is considered to be statistically significant in this exploratory study to identify potential signals. FA, Fractional anisotropy; Dax, axial diffusivity; Drad, radial diffusivity; MD, mean diffusivity; KA, kurtosis anisotropy; AK, axial kurtosis; RK, radial kurtosis; MK, mean kurtosis; AxEAD, axial extra-axonal diffusivity; RadEAD, radial extra-axonal diffusivity, RadIAD, radial intra-axonal diffusivity; AxIAD, axial intra-axonal diffusivity; AWF, axonal water fraction.

The findings are summarized in Figure 3 and detailed in the Supplementary Table 2.

In the anterior segment of CC, Dax and AxEAD were significantly elevated in AD patients as compared to the healthy controls, whereas AK was reduced.

In the mid-anterior segment of CC, a maximum number of diffusion parameters were significantly altered in AD patients as compared to the HCs. The significantly altered parameters were MD, Dax, Drad, and RadEAD- which were significantly higher in AD patients than in controls. In contrast, FA, AK, KA, and AWF were significantly lower in AD patients than in controls.

In the central CC, AK, and MK were lower in AD patients than controls.

The mid-posterior segment of CC showed MD, Drad, and RadEAD that were significantly elevated in AD patients than in controls; while AK, FA, and AWF were significantly lower in AD patients than in controls in this region.

In the posterior segment of CC, MK was significantly lower in AD patients as compared to HCs. No significant changes were found in the other parameters in this region.

#### Correlation of cognitive score with DKI metrics

**Figure 4:**
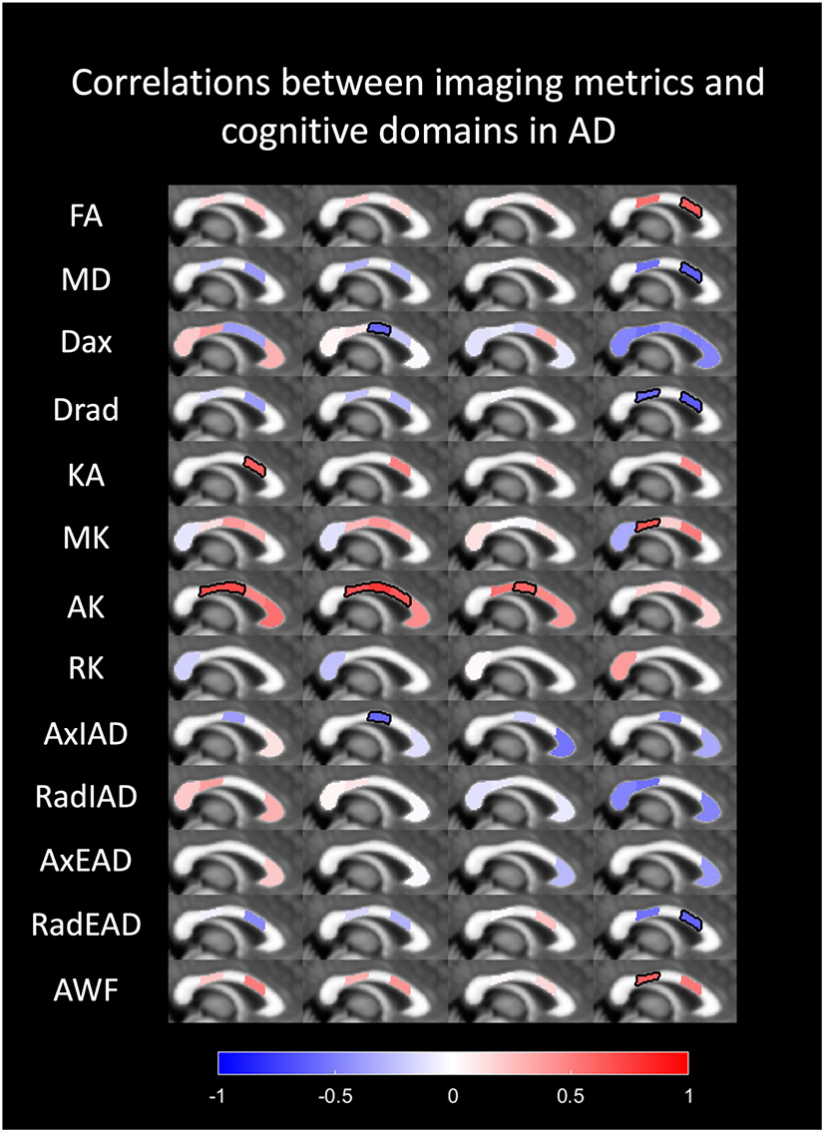
Spearman's correlation between the diffusion parameters and the cognitive scores for the AD patients. Correlation between diffusion metrics and cognitive domains in AD are colored in the respective segments of corpus callosum; red color indicating a positive correlation and blue color indicating a negative correlation. The segments that are outlined in black show statistical significance.

Spearman's correlation between the statistically significant diffusion parameters and the cognitive scores for the AD patients is summarized in Supplementary Table 3 and shown in Figure 4. The correlations that are significant are contoured in black.

We did not find any correlation of the global cognition scores, MMSE and MOCA with regional diffusion metrics. However, impairment of specific cognitive domains in AD correlated with several regional diffusion parameters. The following statistically significant correlations were seen.

The scores for visuospatial function showed a positive correlation with KA in the mid-posterior corpus callosum and AK in the mid-anterior and central segments.

Language function showed a positive correlation with AK in the mid-anterior, central and mid-posterior segments of the callosum and AxIAD in the central segment.

Attention correlated positively with AK in central callosum.

Delayed memory showed a positive correlation with FA in the mid-posterior callosum and a negative correlation with MD and RadEAD in the mid-posterior callosum; negative correlation with Drad in the mid anterior and mid posterior callosal segments, and positive correlation with AWF and MK in the mid-anterior segment.

No correlation of immediate memory with regional diffusion metrics in the CC was seen within our cohort.

## Discussion

We studied the microstructural white matter changes in the segments of the corpus callosum in patients with AD using DTI and the WMTI DKI model and compared it with healthy controls. We found that the corpus callosum of AD patients shows significant diffusion changes compared to HCs. These changes are most prominent in mid-anterior segment, followed by the mid-posterior segment of the corpus callosum.

Among the DTI and DKI metrics we examined, axial kurtosis was the most significantly altered metric, being reduced in AD patients in almost all segments of corpus callosum. Furthermore, reduced axial kurtosis in the CC segments correlated with poor cognition scores in AD patients in the executive, language and attention domains.

### DKI changes in the corpus callosum in Alzheimer's disease

Traditionally, the corpus callosum has been described in the following segments: rostrum, genu, body, isthmus and splenium from the anterior to the posterior direction, although from the imaging perspective no definite visible landmarks define these segments (26). Many geometric and atlas-based techniques have been used to divide the corpus callosal segments (27–30). We used an atlas-based segmentation using Freesurfer wmparc atlas that divides the corpus callosum into 5 equal segments from anterior to posterior and labels them as anterior, mid-anterior, central, mid-posterior and posterior. Regardless of the nomenclature, the anterior and mid-anterior segments of the corpus callosum (which include the rostrum, genu and the proximal body of corpus callosum) contain white matter tracts connecting the frontal and pre-frontal cortices; the central segment (corresponding to the central body of corpus callosum) connects the motor and sensory cortices; the mid-posterior segment connects the somatosensory association cortices; and the posterior most segment (the splenium) connects the visual cortices, the parieto- occipital junctions, the temporal and occipital cortices (31).

Most of the axons that constitute the white matter fibers arise from pyramidal neurons in cortical layers 3 and 5 of the cortex (32). The axons arising from layer 3 form cortico-cortical connections within the same hemisphere and with the contralateral hemisphere (i.e., in the corpus callosum); and axons from layer 5 form cortical-subcortical connections (33). All cortical neurons, but especially the neurons in cortical layers 3 and 5, are affected by the neuritic plaques of AD pathology and the axons undergo Wallerian degeneration following injury to the neurons (34, 35).

The axons are wrapped in layers of myelin sheath, the myelin playing an essential role in increasing the speed of information transfer and information integration in the brain. The myelin in the adult brain is produced, maintained and repaired by oligodendrocytes. Bartzokis suggested that the primary pathology in AD may be dysregulation of the myelin maintenance by the oligodendrocytes as a result of injury due to a variety of insults including ischemic, oxidative, excitotoxic, iron accumulation, amyloid toxicity or taupathy related (36). This leads to myelin loss and demyelination. Theory of retrogenesis, proposes that late myelinating fibers of the anterior corpus callosum are affected earlier by demyelination in the AD process (37, 38). Hence white matter changes in AD may be a result of Wallerian degeneration from cortical neuronal injury or a result of demyelination from injury to oligodendrocytes. In our study, we found evidence of both these processes, most distinctly in the mid-anterior and mid-posterior segments of the corpus callosum of AD patients.

The significantly altered diffusion metrics in the mid-anterior segment in AD patients were increased Drad, increased RadEAD, increased Dax, decreased AK, increased MD, reduced FA, KA, and AWF as compared to the controls. In the mid-posterior segment of AD patients, AWF, AK, and FA were reduced whereas RD, RadEAD and MD were increased. Using these metrics, the pathological processes in the white matter (demyelination and/or Wallerian degeneration) can be interpreted. In general, diffusivity measures depend on the barriers to diffusion of water molecules. In the DTI model, the alteration of the shape and magnitude of the probability of distribution of the diffusion of water molecules is estimated. Water diffuses more freely along the direction of the axons than in another direction, referred to as anisotropic diffusion (39). The direction and orientation of anisotropy is described using eigenvalues and eigenvectors in each voxel (40). However, at b values higher than 1,000 s/mm^2^, diffusion in the brain is observed to be non-Gaussian (41, 42). There are microscopic barriers to diffusion of water such as myelin sheaths, cell membranes, cell organelles, differences in permeability as well as water compartments with varying diffusivity which cause the diffusion distribution probability to be non-Gaussian (14). The DKI model takes into account this non-Gaussian diffusion to compute metrics that are more sensitive to microstructural changes than DTI metrics. Thus, kurtosis measures reflect the microstructural complexity in the tissue and the WMTI model suggests a more specific biophysical profile than DTI measures alone (14, 15). Increased radial diffusivity has been shown to be associated with demyelination whereas axonal degeneration is associated with decreased axial diffusivity in the early/inflammatory phases and increased axial and radial diffusivity are seen with chronic axonal degeneration due to increased tissue isotropy (43, 44). However, these measures are not considered specific (44).

Increased kurtosis measures suggest increased microstructural complexity and when seen in the white matter in AD may be due to glial activation or astrogliosis. Decreased kurtosis suggests loss of tissue complexity and can be seen due to neuronal loss. Axonal water fraction (AWF) refers to the intra-axonal water volume relative to extra-axonal water volume. Decreased AWF is a marker for loss of axonal density such as may occur due to Wallerian degeneration secondary to cortical atrophy (16). Intra-axonal diffusivity (Ax IAD) i.e., the diffusivity within the axons is a marker of axonal injury and is increased in axonal degeneration (16). Diffusivity in the extra axonal space (EAS) increases with demyelination, loss of oligodendrocytes, astrocytosis, with radial extra-axonal diffusivity being more sensitive marker of myelin breakdown (16). Fractional anisotropy, derived from DTI, reflects the anisotropy related to the axonal arrangement/architecture, whereas kurtosis and the other derived metrics are related to the microstructural changes.

Increased diffusivity in the radial direction along with increased radial extra-axonal diffusivity in the mid-anterior corpus callosum in our study, suggests the process of demyelination (43, 45–47). Increased axonal diffusivity along with reduced axial kurtosis and reduced intra axonal water fraction in the mid-anterior segment points toward axonal degeneration (48). This is likely a result of Wallerian degeneration of the commissural fibers secondary to cortical atrophy. Similar changes are observed in the mid-posterior segment of corpus callosum of AD patients, suggesting demyelination and Wallerian degeneration are both prominent pathological processes in these segments of corpus callosum in our study population of AD. The diffusion metrics in the different segments of the corpus callosum may also be influenced by differences in the axonal diameter, axonal packing density, orientation of the fibers in the different segments, affecting the intra and extra axonal spaces.

Many of the earlier DTI studies that evaluated corpus callosal changes in AD patients studied FA and MD and found no significant diffusion changes (12); while several studies found reduced FA in genu and anterior body (49, 50) and splenium (51) of CC. It is difficult to draw conclusions about the biophysical processes responsible for these changes. Subsequent studies have evaluated kurtosis measures, for example, Strufys et al. found decreased FA in the splenium; decreased MK and increased MD in the genu, body and splenium of CC in AD patients as compared to healthy controls (52). Our findings demonstrated differences in some regions compared to their study. We found decreased MK in the mid anterior, central CC (body) and splenium, but we did not find decreased MK in the genu of CC. We found significant reduction in FA in the mid anterior portion of CC rather than the splenium. Fieremans found increase in Rad EAD in the genu of CC in MCI and in the splenium of CC suggesting it is a biomarker of early AD (16, 53–55). Our AD subjects showed increased RAD EAD in the mid anterior and mid posterior CC. A more recent DKI study (56) found a decrease of KA, MK in CC (segments not specified) in AD subjects, similar to our study. Raj et al. reported decrease in AK and RK in the body and splenium of CC in AD subjects (57). Interestingly we found profound reduction in AK in all segments of CC except in the posterior most segment. We did not find significant differences in RK. Some of the disagreements may be explained by differences in the segmentation techniques of the CC. Collij et al. compared DTI measures with amyloid PET imaging using Flumetamol and found that increased amyloid burden correlated with decreased FA, increased mean diffusivity with increased radial diffusivity in the body of corpus callosum (58). Koster et al. found a decrease in the number of myelinated fibers in the anterior segment CC of AD patients on neuropathological studies and suggested that loss of interhemispheric connections may play a role in the pathology of AD (9).

### Correlation of cognitive scores with DKI metrics

Processing of information between neurons in the brain relies on intact connectivity and axonal conduction in the brain circuits. Axonal disruption in the callosal fibers impairs the neural circuitry (59). Higher cognitive tasks such as attention, recall of memory, visuospatial localization and language, require integration of information from the distributed neural systems in both cerebral hemispheres. Injury to connecting tracts results in disconnection and impairment of these functions (60).

We found a significant correlation between the altered diffusion metrics in some segments of corpus callosum in AD patients with specific cognitive domains, although there was no correlation found with the global cognitive scores (MMSE and MOCA). There was a positive correlation of FA in the mid posterior CC with delayed memory, a higher FA in the mid posterior CC was correlated with better performance at memory tasks. A negative correlation of MD, Dax, Drad, and RadEAD in the mid posterior CC with memory was seen in AD patients. Increased Drad in the mid anterior CC was also correlated with worse memory scores. A decrease in kurtosis anisotropy in the mid posterior CC was correlated with a poor score in the visuospatial domain in AD patients. The decrease in the axial kurtosis, which is a marker of axonal degeneration, in the mid anterior, central and mid posterior segments of CC correlated with decreased scores in the visuospatial, language and attention tests.

Although many DTI studies have found correlations between DTI metrics in the brain (mostly FA and MD) with global cognitive measures (61–64), many others have found no correlation (51, 65, 66). Papma et al. found FA changes in the genu of CC correlated more with cerebrovascular diseases rather than with AD pathology (67). Mayo et al. did not find a correlation between DTI metrics (FA, MD, AxD, RD) and cognitive scores in their AD group, although they reported correlation b/w memory and executive dysfunction in a combined sample of HC and AD (68).

We found many of the results that have been reported piecemeal in literature. We have examined multiple DTI and DKI, WMTI metrics in each segment of the CC to get a more comprehensive understanding of the pathophysiology of the callosal changes in AD patients. There are many limitations to our study too, the first one being our small sample size. The small sample size of this study did not allow for multivariable analysis adjusting for the matching factors, hence larger studies are required to validate the findings of this study. Due to the small study group, we did not further stratify our patients by severity into MCI, early and advanced AD. The pathophysiological processes in each of these groups may differ and it limits comparison with other studies in literature. Another limitation is that the diagnosis of AD was made using the NIA-AA criteria, we did not obtain amyloid biomarkers. We used Freesurfer wmparc atlas-based segmentation to subdivide the corpus callosum. Studies in literature have used many different segmentation techniques, some of which been reviewed and published by di Paola et al. (12). Absence of a standardized segmentation method of the CC limits the direct comparison of our results with some of the other studies. Although we found correlation between the altered DKI metrics in corpus callosum and cognitive impairments in specific domains, these changes may not necessarily be due to AD. Other underlying etiologies such as cerebrovascular disease may play an associated role, which have not been investigated in our study.

In conclusion, we explored the biophysical profile of diffusion changes in the CC in AD patients using an array of diffusion metrics with DTI/DKI/WMTI model. We detected changes suggesting demyelination and axonal degeneration throughout the corpus callosum but most prominent in the mid anterior and mid posterior segments of CC. Of all the metrics we examined, we found axial kurtosis to be the most significantly altered metric, being reduced in AD patients in almost all segments of corpus callosum. Reduced axial kurtosis in the CC segments correlated with poor cognition scores in AD patients in the visuospatial, language and attention domains. Increased MD, Dax, Drad, and RadEAD in the posterior central CC correlated with poorer memory scores in AD patients. Further studies are needed to confirm our findings and to evaluate the use of axial kurtosis in the mid anterior, central and mid posterior segments of CC as a biomarker of AD.

## Data availability statement

The raw data supporting the conclusions of this article will be made available by the authors, without undue reservation.

## Ethics statement

The studies involving human participants were reviewed and approved by SingHealth Centralized Institutional Review Board (CIRB). The patients/participants provided their written informed consent to participate in this study.

## Author contributions

SK and NK: concept and design, administrative and take responsibility for the integrity of the data and the accuracy of the data analysis. FZ: project administration and performed the cognitive tests. AD and AL: design of MRI acquisition, analysis and interpretation of data, and drafting of the manuscript. SK, SH, and NK: interpretation of data and drafting of the manuscript. SS: statistical analysis and drafting of the manuscript. All authors contributed to the article and approved the submitted version.

## Funding

This study was supported by SingHealth Duke-NUS Radiological Sciences Academic Clinical Programme and by the National Medical Research Council (NMRC), Singapore, under its Clinician Sciences Award (MOH-CSAINV18nov-0007).

## Conflict of interest

The authors declare that the research was conducted in the absence of any commercial or financial relationships that could be construed as a potential conflict of interest.

## Publisher's note

All claims expressed in this article are solely those of the authors and do not necessarily represent those of their affiliated organizations, or those of the publisher, the editors and the reviewers. Any product that may be evaluated in this article, or claim that may be made by its manufacturer, is not guaranteed or endorsed by the publisher.
