# Corrigendum: Topology of diffusion changes in corpus callosum in Alzheimer's disease: An exploratory case-control study

**DOI:** 10.3389/fneur.2023.1188454

**Published:** 2023-03-30

**Authors:** Sumeet Kumar, Alberto De Luca, Alexander Leemans, Seyed Ehsan Saffari, Septian Hartono, Fatin Zahra Zailan, Kok Pin Ng, Nagaendran Kandiah

**Affiliations:** ^1^National Neuroscience Institute, Singapore, Singapore; ^2^Duke-NUS Graduate Medical School, Singapore, Singapore; ^3^Image Sciences Institute, UMC Utrecht, Utrecht, Netherlands; ^4^Lee Kong Chian School of Medicine, Nanyang Technological University, Singapore, Singapore

**Keywords:** Alzheimer's disease, diffusion tensor imaging, white matter tract integrity, corpus callosum, magnetic resonance imaging (MRI), diffusion kurtosis imaging, axial kurtosis

In the published article, there was an error in the Funding statement. The funding from the National Medical Research Council (NMRC) was mistakenly omitted. The correct Funding statement appears below.

