# Supplementary material for: Topology of diffusion changes in corpus callosum in Alzheimer's disease: An exploratory case-control study
Source: Front Neurol. 2022 Nov 30;13:1005406. doi: 10.3389/fneur.2022.1005406 (PMC9747939; doi:10.3389/fneur.2022.1005406)
Supplement: Supplementary file 1 [file Data_Sheet_1.PDF]

Supplementary Table 1. Fazekas score of the study participants

Subject	Periventricular	Deep white matter
A01	3	2
A02	3	1
A03	3	2
A04	1	1
A05	3	3
A06	1	1
A07	2	1
A08	1	1
A09	1	1
A10	1	2
A11	1	1
A12	1	1
C01	1	1
C02	1	0
C03	1	1
C04	1	1
C05	1	1
C06	2	2

C07	2	1
C08	2	1
C09	1	1
C10	1	0
C11	1	1
C12	2	2

Note: Periventricular white matter: 0 = absent, 1 = “caps” or pencil-thin lining, 2 = smooth “halo”, 3 = irregular periventricular signal extending into the deep white matter; Deep white matter: 0 = absent, 1 = punctate foci, 2 = beginning confluence, 3 = large confluent areas. [reference: Fazekas AJR 1987]

Supplementary Table 2. Comparison of all DTI/DKI indices between AD and HC groups

Region	Index	AD mean (SD)	HC mean (SD)	p
CC anterior	MD	1.63 (0.22)	1.47 (0.18)	0.140
	Dax	2.25 (0.39)	1.86 (0.33)	0.052
	Drad	1.32 (0.22)	1.27 (0.15)	0.684
	FA	0.37 (0.13)	0.27 (0.10)	0.141
	AxEAD	2.96 (0.53)	2.43 (0.47)	0.053
	RadEAD	1.72 (0.24)	1.62 (0.18)	0.389
	AxIAD	0.61 (0.23)	0.42 (0.15)	0.083
	RadIAD	0.13 (0.04)	0.10 (0.03)	0.115
	AWF	0.30 (0.06)	0.27 (0.05)	0.325
	MK	0.84 (0.13)	0.96 (0.20)	0.192
	AK	0.74 (0.08)	0.81 (0.03)	0.055
	RK	0.83 (0.24)	0.73 (0.26)	0.439
	KA	0.23 (0.10)	0.19 (0.08)	0.361
CC mid-anterior	MD	1.67 (0.19)	1.42 (0.17)	0.027
	Dax	2.46 (0.21)	2.21 (0.27)	0.070
	Drad	1.27 (0.19)	1.03 (0.14)	0.023
	FA	0.44 (0.05)	0.51 (0.03)	0.008
	AxEAD	3.22 (0.24)	3.08 (0.29)	0.322

	RadEAD	1.71 (0.21)	1.45 (0.15)	0.019
	AxIAD	0.78 (0.09)	0.72 (0.14)	0.337
	RadIAD	0.12 (0.03)	0.10 (0.02)	0.164
	AWF	0.33 (0.04)	0.39 (0.03)	0.011
	MK	0.91 (0.10)	1.04 (0.09)	0.030
	AK	0.68 (0.06)	0.81 (0.08)	0.012
	RK	1.09 (0.18)	1.00 (0.66)	0.692
	KA	0.27 (0.07)	0.38 (0.09)	0.029
CC central	MD	1.55 (0.14)	1.48 (0.19)	0.428
	Dax	2.41 (0.17)	2.26 (0.24)	0.199
	Drad	1.12 (0.14)	1.08 (0.18)	0.692
	FA	0.50 (0.05)	0.49 (0.05)	0.842
	AxEAD	3.23 (0.22)	3.07 (0.28)	0.246
	RadEAD	1.57 (0.17)	1.52 (0.21)	0.610
	AxIAD	0.90 (0.10)	0.78 (0.13)	0.062
	RadIAD	0.11 (0.03)	0.10 (0.02)	0.231
	AWF	0.36 (0.03)	0.37 (0.04)	0.739
	MK	0.97 (0.08)	1.06 (0.08)	0.052
	AK	0.66 (0.04)	0.73 (0.04)	0.008
	RK	1.29 (0.20)	1.33 (0.18)	0.702
	KA	0.35 (0.07)	0.35 (0.10)	0.990

CC mid-posterior	MD	1.67 (0.29)	1.39 (0.27)	0.060
	Dax	2.50 (0.25)	2.26 (0.34)	0.141
	Drad	1.25 (0.31)	0.95 (0.25)	0.059
	FA	0.46 (0.09)	0.55 (0.06)	0.047
	AxEAD	3.30 (0.23)	3.14 (0.36)	0.305
	RadEAD	1.72 (0.34)	1.38 (0.29)	0.060
	AxIAD	0.93 (0.11)	0.90 (0.13)	0.656
	RadIAD	0.12 (0.03)	0.09 (0.01)	0.073
	AWF	0.35 (0.06)	0.40 (0.05)	0.082
	MK	0.94 (0.16)	1.06 (0.10)	0.121
	AK	0.62 (0.04)	0.70 (0.04)	0.009
	RK	1.31 (0.33)	1.53 (0.21)	0.144
	KA	0.35 (0.14)	0.43 (0.11)	0.229
CC posterior	MD	1.64 (0.11)	1.56 (0.17)	0.313
	Dax	2.57 (0.16)	2.46 (0.18)	0.225
	Drad	1.17 (0.14)	1.11 (0.18)	0.491
	FA	0.51 (0.07)	0.52 (0.06)	0.684
	AxEAD	3.46 (0.26)	3.35 (0.20)	0.358
	RadEAD	1.64 (0.16)	1.58 (0.21)	0.480
	AxIAD	0.99 (0.12)	0.95 (0.09)	0.470
	RadIAD	0.12 (0.03)	0.10 (0.01)	0.090

	AWF	0.37 (0.04)	0.39 (0.04)	0.433
	MK	0.92 (0.13)	1.06 (0.08)	0.030
	AK	0.64 (0.05)	0.66 (0.03)	0.172
	RK	1.07 (0.71)	1.54 (0.17)	0.110
	KA	0.39 (0.11)	0.42 (0.09)	0.536

Supplementary Table 3. Correlation between imaging metrics and cognitive domains in AD patients

Variable	VISUOSPATIAL		LANGUAGE		ATTENTION		DELAYED_MEMORY	
	Correlation	p value	Correlation	p value	Correlation	p value	Correlation	p value
AK_CC_Mid_Posterior	0.48	0.1144	0.65	0.0221	0.46	0.1287	0.33	0.3021
AK_CC_Mid_Anterior	0.69	0.0130	0.66	0.0184	0.55	0.0649	0.20	0.5369
FA_CC_Mid_Posterior	0.24	0.4492	0.15	0.6453	0.11	0.7443	0.64	0.0251
AK_CC_Central	0.70	0.0112	0.77	0.0031	0.60	0.0373	0.17	0.5929
RadEAD_CC_Mid_Posterior	-0.46	0.1335	-0.30	0.3428	0.24	0.4544	-0.60	0.0383
RD_CC_Mid_Anterior	-0.39	0.2334	-0.29	0.3797	0.00	1.0000	-0.64	0.0338
KA_CC_Mid_Posterior	0.62	0.0303	0.49	0.1049	0.17	0.5925	0.46	0.1324
MD_CC_Mid_Posterior	-0.40	0.2275	-0.29	0.3876	0.08	0.8101	-0.61	0.0477
RD_CC_Mid_Posterior	-0.14	0.6721	-0.25	0.4315	-0.04	0.8964	-0.59	0.0431
AxIAD_CC_Central	-0.38	0.2291	-0.58	0.0495	-0.19	0.5614	-0.45	0.1426
AWF_CC_Mid_Anterior	0.22	0.4837	0.30	0.3369	0.03	0.9223	0.62	0.0299
MK_CC_Mid_Anterior	0.13	0.6881	0.28	0.3859	-0.01	0.9741	0.66	0.0200
