# Supplementary figures and images for: Topology of diffusion changes in corpus callosum in Alzheimer's disease: An exploratory case-control study
Source: Front Neurol. 2022 Nov 30;13:1005406. doi: 10.3389/fneur.2022.1005406 (PMC9747939; doi:10.3389/fneur.2022.1005406)

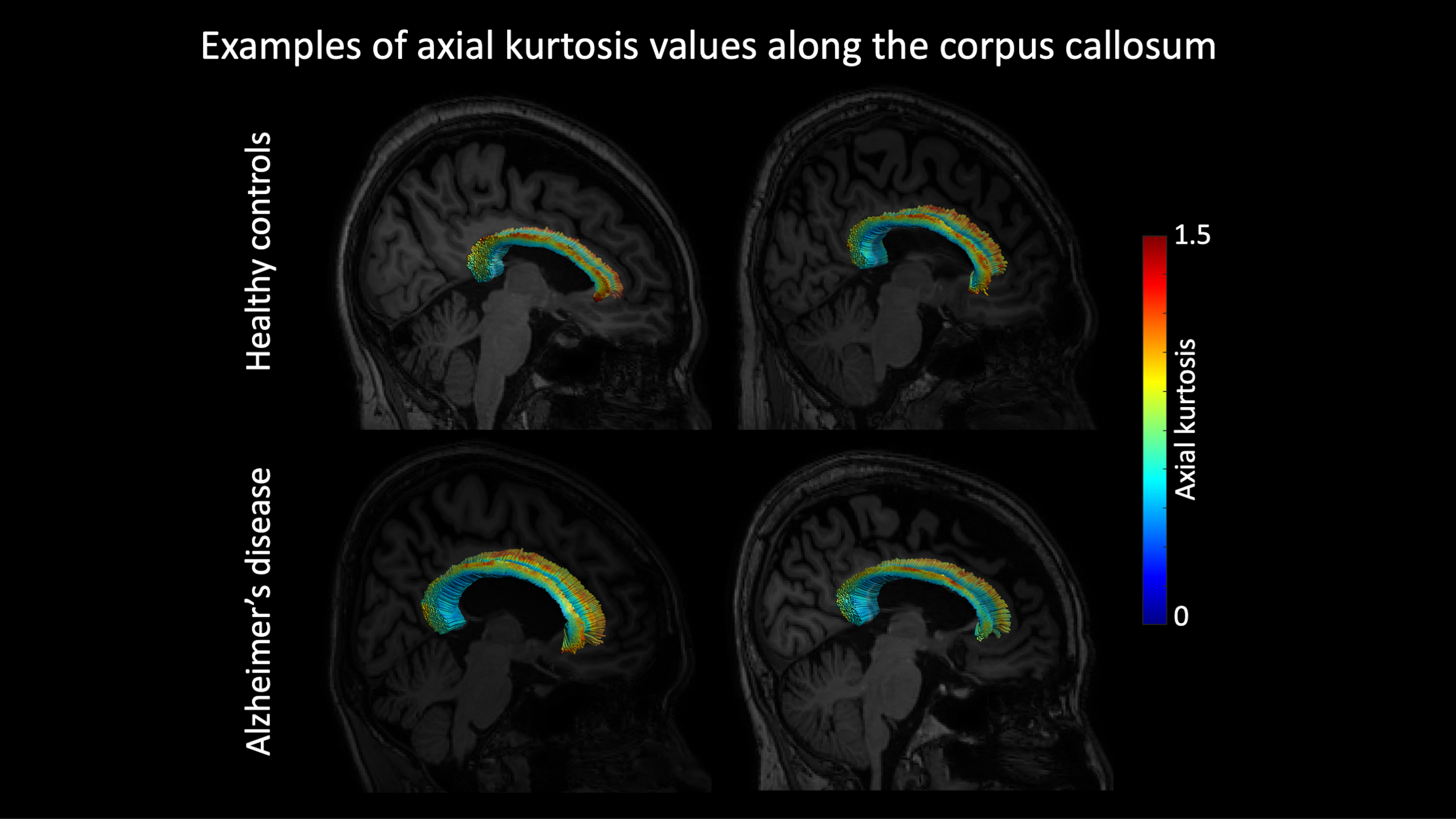

Supplement: Supplementary file 2 [file Image_1.TIFF]
