# Supplementary material for: Topology of diffusion changes in corpus callosum in Alzheimer's disease: An exploratory case-control study
Source: Front Neurol. 2022 Nov 30;13:1005406. doi: 10.3389/fneur.2022.1005406 (PMC9747939; doi:10.3389/fneur.2022.1005406)
Supplement: Supplementary file 3 [file Image_2.pdf]

Supplementary Figure 2. Voxel-wise t-tests for each considered metric were performed using the FSL TBSS pipeline. In short, the FA image of each subject was registered to MNI space with a combination of linear (FSL FLIRT) and non-linear (FSL FNIRT) transformations. The same transformations were applied to all other considered metrics. Subsequently, t-tests were performed using FSL randomise, considering 1000 permutation tests and threshold free cluster enhancement (TFCE). The following are the VBM results shown on the sagittal (a) and coronal(b) planes, 2 tests per map, green colour indicating the significant voxels. Legend: FA (fractional anisotropy), Dax (axial diffusivity), Drad (radial diffusivity), MD (mean diffusivity), KA (kurtosis anisotropy), RK (radial kurotsis), AK (axial kurtosis), MK (mean kurtosis), AxEAD (axial extra axonal diffusivity), RadEAD (radial extra axonal diffusivity), AxIAD (axial intra axonal diffusivity), RadIAD (radial intra axonal diffusivity), AWF (axonal water fraction).

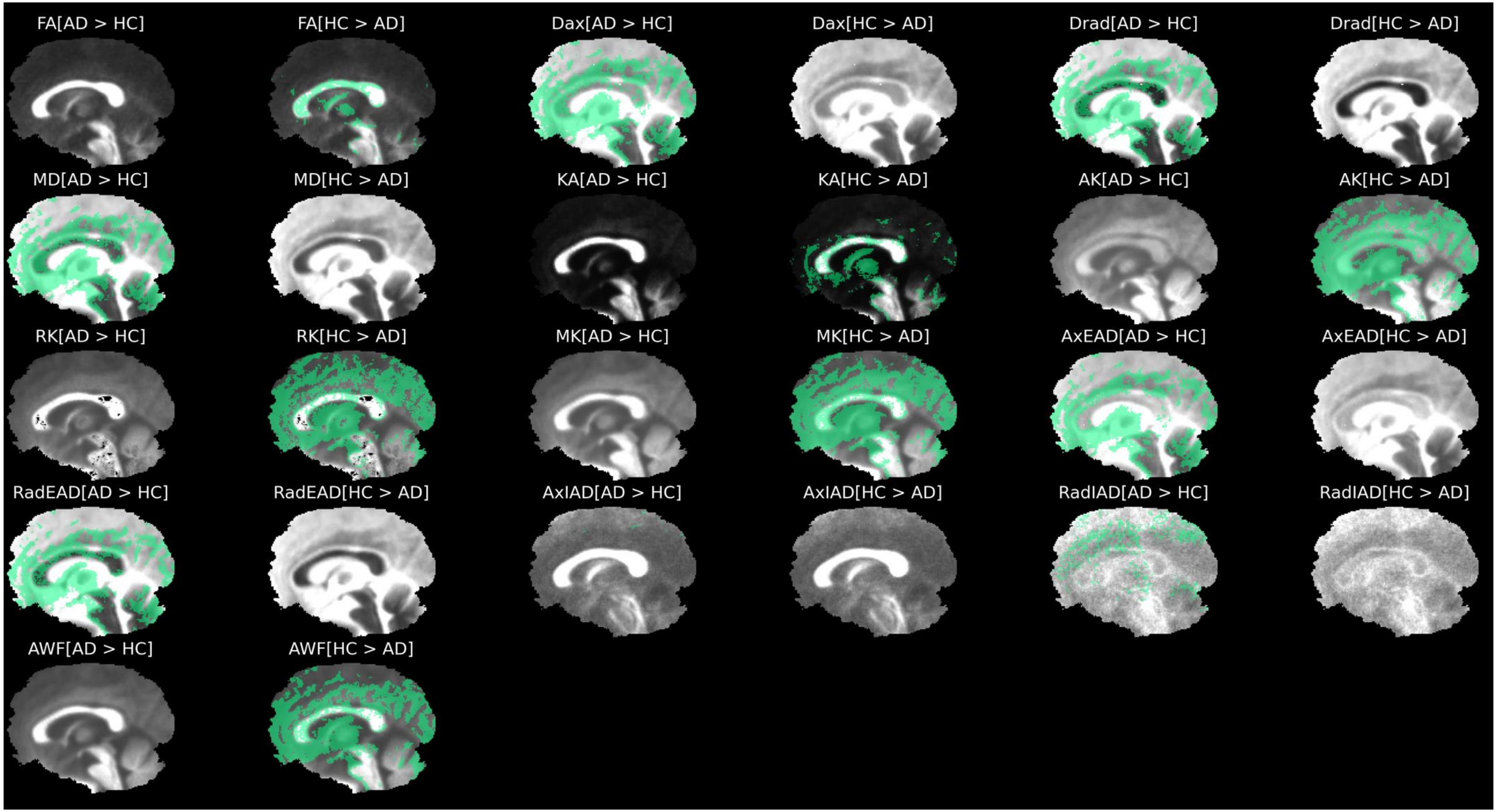
FA[AD > HC]
FA[HC > AD]
Dax[AD > HC]
Dax[HC > AD]
Drad[AD > HC]
Drad[HC > AD]
MD[AD > HC]
MD[HC > AD]
KA[AD > HC]
KA[HC > AD]
AK[AD > HC]
AK[HC > AD]
RK[AD > HC]
RK[HC > AD]
MK[AD > HC]
MK[HC > AD]
AxEAD[AD > HC]
AxEAD[HC > AD]
RadEAD[AD > HC]
RadEAD[HC > AD]
AxIAD[AD > HC]
AxIAD[HC > AD]
RadIAD[AD > HC]
RadIAD[HC > AD]
AWF[AD > HC]
AWF[HC > AD]

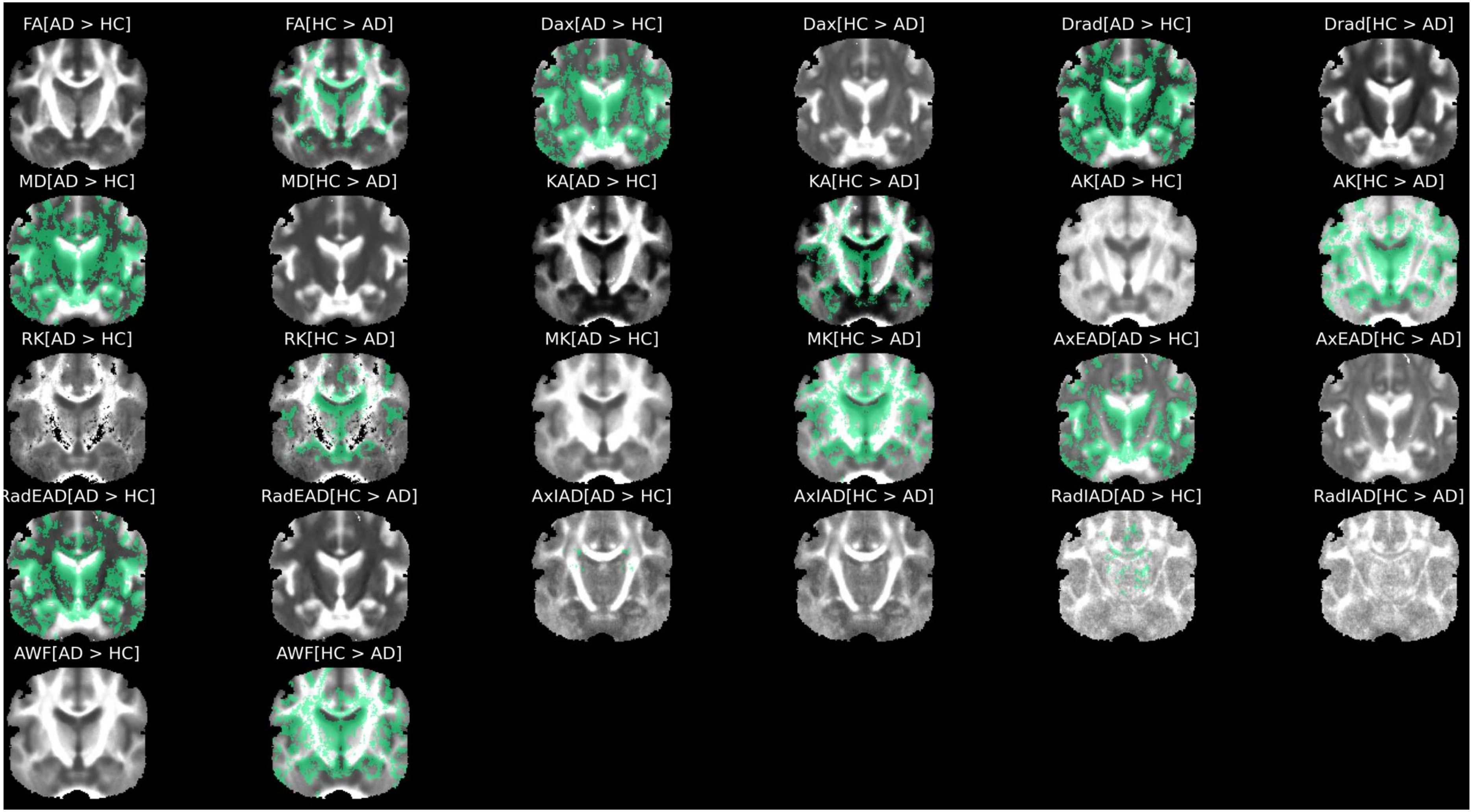
FA[AD > HC]
FA[HC > AD]
Dax[AD > HC]
Dax[HC > AD]
Drad[AD > HC]
Drad[HC > AD]
MD[AD > HC]
MD[HC > AD]
KA[AD > HC]
KA[HC > AD]
AK[AD > HC]
AK[HC > AD]
RK[AD > HC]
RK[HC > AD]
MK[AD > HC]
MK[HC > AD]
AxEAD[AD > HC]
AxEAD[HC > AD]
RadEAD[AD > HC]
RadEAD[HC > AD]
AxIAD[AD > HC]
AxIAD[HC > AD]
RadIAD[AD > HC]
RadIAD[HC > AD]
AWF[AD > HC]
AWF[HC > AD]
